# Early identification of golimumab-treated patients with higher likelihood of long-term retention

**DOI:** 10.3389/fimmu.2024.1359571

**Published:** 2024-04-05

**Authors:** Alicia García-Dorta, Enrique González-Dávila, Marta Sánchez-Jareño, Luis Cea-Calvo, Manuel Pombo-Suárez, Fernando Sánchez-Alonso, Isabel Castrejón, Federico Díaz-González

**Affiliations:** ^1^Departamento de Reumatología, Hospital Universitario de Canarias, Santa Cruz de Tenerife, Spain; ^2^Departamento de Matemáticas, Estadística e Investigación Operativa, Instituto de Matemáticas y Aplicaciones de la Universidad de La Laguna (IMAULL), Universidad de La Laguna, Santa Cruz de Tenerife, Spain; ^3^Medical Affairs, MSD Spain, Madrid, Spain; ^4^Departamento de Reumatología, Complejo Hospital Universitario de Santiago de Compostela, La Coruña, Spain; ^5^Unidad de Investigación de la Sociedad Española de Reumatología (UI-SER), Madrid, Spain; ^6^Departamento de Reumatología, Instituto de Investigación Sanitaria Gregorio Marañón (IiSGM), Hospital Universitario Gregorio Marañon, Universidad Complutense de Madrid, Madrid, Spain; ^7^Departamento de Medicina Interna, Dermatología y Psiquiatría, Universidad de La Laguna, Santa Cruz de Tenerife, Spain; ^8^Instituto de Tecnologías Biomédicas (ITB), Universidad de La Laguna, Santa Cruz de Tenerife, Spain

**Keywords:** golimumab, treatment retention, rheumatoid arthritis, axial spondyloarthritis, psoriatic arthritis, biological therapy

## Abstract

**Background:**

The early identification of patients’ profiles most likely to respond to and maintain long-term therapy with a biological drug can have clinical and cost-effectiveness implications.

**Objectives:**

To evaluate the utility of an innovative approach for early identification of patient profiles associated with long-term persistence of golimumab, a tumour necrosis factor inhibitor, in patients with rheumatoid arthritis (RA), psoriatic arthritis (PsA), and axial spondyloarthritis (SpA) under real-world conditions.

**Design:**

Retrospective non-interventional database analysis.

**Methods:**

Kaplan-Meier curves of golimumab retention over 8 years from the BIOBADASER registry, overall and by indication, were analysed using a novel approach (a two-phase decay model) to identify the point at which the golimumab retention curve shifted from rapid (indicating high golimumab discontinuation rate) to slow decay (low discontinuation rate). Factors associated with golimumab retention at these time points were identified using Cox regression, and retention rates for different patient profiles were calculated.

**Results:**

885 patients were included. The golimumab retention curve shifted from rapid to slow decay at month 10 for the overall population (retention rate: 73.4%), at month 24 for RA patients (retention: 45.0%), and at month 8 for SpA, including axial SpA and PsA (81.6%). Factors associated with golimumab discontinuation at these early points were, overall, similar to those previously identified at year 8 (RA diagnosis, golimumab as second- or third-line of biological therapy, disease activity over the median and treatment with corticosteroids at golimumab initiation, advanced age [in RA], and female gender [in SpA]).

**Conclusion:**

With this novel approach, the factors associated with long-term retention were identified in the initial period of rapid discontinuation of golimumab.

## 1 Introduction

Tumour necrosis factor inhibitors (TNFi) are biological disease-modifying antirheumatic drugs (DMARDs) indicated for the treatment of patients with immune-mediated diseases (1), including several rheumatic diseases (2–4). In general, biologic long-term treatment is required to achieve maximum clinical benefit; therefore, the selection of patients in whom there is a reasonably high probability of long-term retention of biological therapy is important from a clinical and cost-effectiveness perspective (5–7). However, patients treated with biologics, including TNFi, do not all respond equally or maintain their response over time and discontinue treatment for various reasons, primarily lack or loss of efficacy, poor tolerability, or adverse events (8–11).

When analysing long-term TNFi retention curves from patients with rheumatoid arthritis (RA) and spondyloarthritis (SpA) under real-life conditions, an early accelerated loss (high rate of TNFi discontinuation) followed by a period of sustained retention (low rate of TNFi discontinuation) is often observed (10–13). Considering this consistent pattern, it is reasonable to assume that knowledge of the profile of patients who are more likely to persist on a particular biological therapy early in treatment may help guide the selection of the most appropriate biological drug.

Golimumab is a biological TNFi indicated for the treatment of RA and SpA including axial spondyloarthritis (ax-SpA) and psoriatic arthritis (PsA) (14). Phase 3 clinical trial extension studies have shown a high long-term golimumab retention rate of up to 5 years (15, 16) as first-line biological drug. In previous work in routine clinical practice, we retrospectively studied the retention rates of golimumab (10). We described 8-year golimumab retention rates of 24.6%, 45.8% and 39.9% in RA, ax-SpA and PsA respectively. Variables associated with discontinuation differed between indications: in RA patients, older age, line of therapy, treatment with glucocorticoid treatment and high disease activity at golimumab initiation were associated with golimumab discontinuation, while in ax-SpA patients, variables associated with discontinuation were female sex, line of therapy and high disease activity, and line of therapy was associated in PsA patients. However, the retrospective nature of the analysis (performed after golimumab had been available for more than 8 years) can limit its applicability in routine clinical practice. Identifying these variables related to long-term persistence earlier (i.e., 2 or 3 years after entering the market), can help to better select patients for specific high-cost therapies.

This study assessed whether the patient profile associated with an increased likelihood of golimumab retention (drug survival or persistence) in the long term could be identified early. To find an early time for the prediction of long retention rate, the Kaplan-Meier curve of golimumab retention over 8 years in patients with RA and SpA was analysed using a two-stage exponential decay equation to determine the time at which the retention curve shifted from fast decay (denoting a high rate of golimumab discontinuation) to slow decay (low rate of golimumab discontinuation). During the rapid decay period, diagnostic and patient disease and treatment characteristics associated with golimumab discontinuation were analysed to assess potential impact on long-term persistence of golimumab. A further objective was to explore differences in RA and SpA regarding time at which the retention curve shifted from fast to slow decay and factors associated with early discontinuation of golimumab. This type of early testing may have important clinical and cost-effectiveness implications for the selection of biologics.

## 2 Methods

This retrospective, non-interventional analysis of golimumab retention was carried out using the BIOBADASER database, the Spanish registry of biological drugs. The main characteristics of the registry have been described elsewhere previously (17). For this analysis, all adults enrolled in BIOBADASER who had started a first cycle of golimumab for RA or SpA (including ax-SpA and PsA) treatment, more than 6 months before the date of data extraction (November 2021) were included. Covariates included sex, age, disease duration at golimumab initiation, diagnosis, line of biological therapy, co-treatment with methotrexate and/or glucocorticoids, and disease activity as assessed by DAS28 erythrocyte sedimentation rate (RA, PsA) (18) or BASDAI (ax-SpA) (19).

All procedures and materials complied with the principles of the Declaration of Helsinki and with Spanish regulations on data protection and research. Patients provide informed consent prior to enrolling in BIOBADASER, which includes consent for subsequent analysis of anonymised aggregated data, as approved by the Clinical Research Committee of the Hospital Universitario Clinic Barcelona (code FER-ADA-2015-01). Consequently, specific informed consent for the current analysis was not needed.

### 2.1 Statistical analysis

Descriptive statistics are displayed as means with standard deviations (SD), medians with interquartile ranges (IQR), or percentages (%). Golimumab retention was defined as the probability of long-term drug retention of up to 8 years’ treatment and was assessed using Kaplan–Meier survival analysis as described elsewhere (10). The observed retention curve of golimumab was fitted to a two-phase exponential decay curve using GraphPad Prism version 10.0.0 for Windows, GraphPad Software, Boston, Massachusetts USA, www.graphpad.com.

The double exponential fitting model combines two exponential functions, one fast and one slow. The model is defined by the following formula:


$$\text{Retention}\left(\text{t}\right)={\text{SpanFast}\;}^{*}\;\text{exp}\left(-\;{\text{KFast}\;}^{*}\text{ t}\right)+\;{\text{SpanSlow}\;}^{*}\text{ exp }(-\;{\text{KSlow}\;}^{*}\text{ t}).$$

Retention (t) is the retention assessed at t (time) and “t” represents the independent variable, SpanFast and SpanSlow stand for the amplitude of the fast and slow exponential components, respectively, and KFast and KSlow represent the inverse of the time constant of the fast and slow exponential components, respectively. Fitting the data to this model enables estimation of the optimal values for SpanFast, SpanSlow, KFast, and KSlow, using a nonlinear fit to maximise the coefficient of determination (R^2^).

The retention rate value at the point of transition of the curve from fast to slow decay (i.e., from high to slow golimumab discontinuation rate) was calculated according to the following formula:


$$\text{Retention }\left(\text{transition from fast to slow}\right)\;={\text{ Retention}}_{0}-\;\left({\text{Retention}}_{0}-\text{ Plateau}\right){\;}^{*}\;{\text{ PercentFast}\;}^{*}\;0.01$$

where Retention_0_ is the retention value when X(time) is zero, Plateau is the retention value at infinite times and PercentFast is the fraction of the span (from Retention_0_ to Plateau) where the decay is fastest (defined as (SpanFast/[SpanFast + SpanSlow])*100). No predefined constants were included in the model.

After identifying the time point when the retention curve showed a change in trend (i.e., when golimumab discontinuation changed from high to low discontinuation rate), the variables associated with the retention rate at that moment were analysed using Cox regression analysis. The Cox-regression model was built with the forward Wald variable selection method on those variables that showed p<0.2 in the univariate analyses. The median of activity indexes (DAS 28 and BASDAI) was used to include this variable in the model as binary (above or below the median). The 2-factors interactions were also analysed using a multivariable Cox proportional hazards regression model during the fast decay period for the total population. Hazard ratios (HR) for golimumab discontinuation and 95% confidence intervals (95% CI) were calculated. Analyses were performed for the overall population, and separately for the RA and the SpA (including ax-SpA and PsA) cohorts. Pooling of patients with ax-SpA and PsA was performed since the Kaplan–Meier curves overlapped and showed similar shape and retention rates.

Finally, with these variables identified, the retention rates for subgroups defined by combinations of the different variables at the timepoint the retention curve changed from fast to slow decay were calculated. All statistical analyses were performed using SPSS (version 25, IBM SPSS, Armonk, NY). Results were considered statistically significant if p< 0.05.

## 3 Results

The study included 885 patients who received a first treatment with golimumab in any line of biological therapy, of whom 473 were women (53.5%) and 412 were men (46.6%). **Table 1** summarises the patients’ baseline demographic and clinical characteristics, disease activity, and concomitant medications at golimumab initiation. By diagnosis, there were 267 (30.2%) patients with RA and 618 (69.8%) with SpA, of whom 370 (41.8%) had ax-SpA (330 radiographic and 40 non-radiographic forms) and 248 (28.0%) had PsA. The mean disease duration since diagnosis for all patients included in the study was 7.6 years (IQR 2.8 - 14.4).

**Table 1:** Characteristics of the study population at golimumab initiation.

	RA (n=267)	Spondyloarthritis (n=618)			All patients (n=885)
		Ax-SpA (n=370)	PsA (n=248)	All SpA	
**Age,** years, mean (SD)	57.4 (12.2)	48.2 (12.3)	50.2 (11.6)	50.0 (12.1)	51.5 (12.7)
**Female**, n (%)	205 (76.8)	130 (35.1)	138 (55.7)	268 (43.4)	473 (53.5)
Disease duration*, years, median (IQR)	8.5 (3.5 – 14.6)	7.3 (2.3 – 16.0)	7.1 (3.0 – 12.5)	7.2 (2.5 – 14.4)	7.6 (2.8 – 14.4)
Smoking habit, n (%)					
Never	152 (56.9)	191 (51.6)	159 (64.1)	350 (56.6)	502 (56.7)
Current	51 (19.1)	113 (30.5)	47 (19.0)	160 (25.9)	211 (23.8)
Past	47 (17.6)	43 (11.6)	29 (11.7)	72 (11.7)	119 (13.5)
Not available	17 (6.4)	23 (6.2)	13 (5.2)	36 (5.8)	53 (6.0)
BMI, n (%)					
<25 kg/m^2^ (Normal weight)	77 (28.8)	89 (24.1)	60 (24.2)	149 (24.1)	226 (25.5)
25 to<30 kg/m^2^ (Overweight)	85 (31.8)	125 (33.8)	84 (33.9)	209 (33.8)	294 (33.2)
≥30 kg/m^2^ (Obesity)	62 (23.2)	84 (22.7)	62 (25.0)	146 (23.6)	208 (23.5)
Not available	43 (16.1)	72 (19.5)	42 (16.9)	114 (18.4)	157 (17.7)
Disease activity indexes					
DAS28 (median, IQR)	4.6 (3.5 – 5.4)	–	4.1 (3.0 – 5.0)	–	–
BASDAI (median, IQR)	–	6.0 (4.5 – 7.3)	5.2 (3.0 – 6.7)	5.8 (4.3 – 7.2)	–
Biomarkers of inflammation					
C-Reactive Protein, mg/L, median (IQR)	2.3 (0.6 – 7.3)	2.5 (1.0 – 5.0)	2.3 (0.9 – 7.0)	2.4 (0.9 – 6.9)	2.3 (0.8 – 7.0)
Erythrocyte sedimentation rate, mm/hour, median (IQR)	22 (10 – 41)	13 (6 – 33)	18 (7 – 33)	16 (7 – 33)	18 (8 – 35)
Medication at golimumab initiation					
Methotrexate, n (%)	138 (51.7)	53 (14.3)	103 (41.5)	156 (25.2)	294 (33.2)
Glucocorticoids, n (%)	153 (57.3)	38 (10.3)	64 (25.8)	102 (16.5)	255 (28.8)

*Disease duration: time since diagnosis to golimumab initiation.

Ax-SpA, axial spondyloarthritis; BASDAI, Bath Ankylosing Spondyloarthritis Disease Activity Index; BMI, body mass index; DAS, Disease Activity Score; IQR, interquartile range; PsA, psoriatic arthritis; RA, rheumatoid arthritis; SD, standard deviation; SpA, spondyloarthritis.

### 3.1 Assessment of the time point of the trend change in the retention rate of golimumab

**Figure 1:**
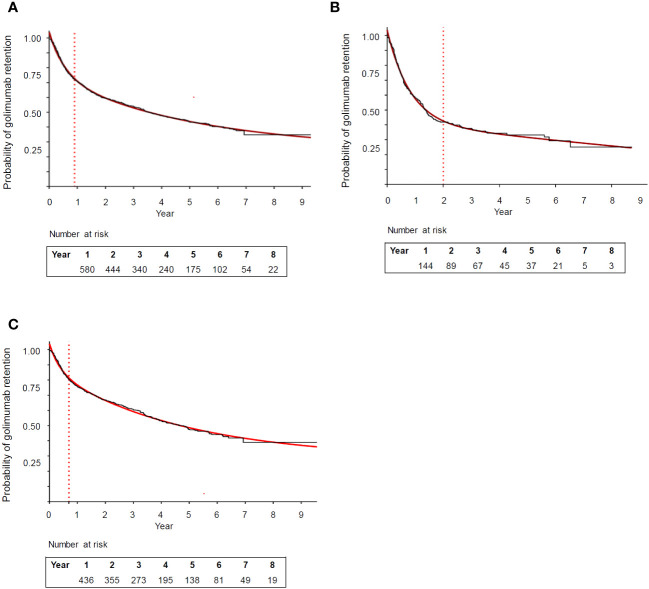
Retention rate curve of golimumab in the: **(A)** overall population (rheumatoid arthritis, axial spondyloarthritis, psoriatic arthritis) **(B)** rheumatoid arthritis, and **(C)** spondyloarthropathies (axial spondyloarthritis plus psoriatic arthritis) subgroup. The red line represents the fit of the Kaplan-Meier retention curves to two-phase exponential decay curves (R^2^ = 0.99 for each curve). The dotted line identifies the timepoint the curve changes from fast to slow decay.

Because the Kaplan-Meier retention curves differed between the general population, RA, PsA, and ax-SpA patients, but not between the last two indications among themselves, analyses were performed for the overall population and separately for RA and SpA (including PsA and ax-SpA). The Kaplan-Meier curves of the overall population (**Figure 1A**), RA (**Figure 1B**), and SpA (ax-SpA plus PsA) patients (**Figure 1C**) were fitted to a two-phase exponential decay curve showing an excellent coefficient of determination (R^2^) >0.99 in the three fits. For the overall population, month 10 was identified as the time when the curve changed from fast to slow decay (i.e., from high to low golimumab discontinuation rate, **Figure 1A**). The retention rate of golimumab at this time point was 73.4% (95% CI 70.5 - 76.3). The same analysis in RA patients identified month 24 as the time when the curve changed from fast to slow decay (**Figure 1B**), the retention rate at that moment being 45.0% (95% CI 38.7 - 51.3). For SpA (ax-SpA plus PsA), the change in the trend was identified at month 8 (**Figure 1C**), and the retention rate at that time was 81.6% (95% CI 78.5 - 84.7). The respective 8-year retention rates for the overall population and for the RA and SpA subgroups were 37.7% (95% CI 33.3 - 42.1), 24.6% (95% CI 15.4 - 35.0), and 41.9% (95% CI 36.6 - 47.2).

### 3.2 Factors associated with golimumab retention in the initial fast decay

The analysis of factors associated with golimumab retention during the initial rapid decline period was carried out separately in the total population, in RA and in SpA patients.

#### 3.2.1 Overall population

In the univariate analysis for the entire population (**Table 2**), the golimumab retention rate during the initial rapid decline period (at month 10) was significantly higher in men, in patients with SpA, in younger patients (<45 years), when golimumab was used as first line of biological therapy, in patients not using glucocorticoids, and in those with disease activity below the median at golimumab initiation. Cox regression analysis (**Table 3**), showed that factors associated with the discontinuation of golimumab at month 10 were the diagnosis of RA compared with SpA (HR 1.98, 95% CI 1.43 - 2.74, p<0.001), the use of golimumab in second line (HR 1.75, 95% CI 1.19 - 2.58, p=0.005) or third line (HR 2.44, 95% CI 1.69 - 3.53, p<0.001) compared to first line of biological therapy, disease activity higher than the median (HR 1.44, 95% CI 1.07 - 1.92, p=0.015), and the use of glucocorticoids at golimumab initiation (HR 1.40, 95% CI 1.00 - 1.93, p=0.048).

**Table 2:** Univariate analysis. Retention rates when the curve changed from fast to slow decay, for the total (month 10), RA (month 24), and SpA (month 8) populations.

		Overall		RA		SpA*	
		Retention rate (95% CI)	p	Retention rate (95% CI)	p	Retention rate (95% CI)	p
**Gender**	Men	80.5 (76.6 – 84.4)	<0.001	46.5 (33.8 – 59.2)	0.473	86.0 (82.3 – 89.7)	0.001
	Women	67.2 (62.9 – 71.5)		41.1 (34.0 – 48.2)		75.7 (70.6 – 80.8)	
**Diagnosis**	RA	61.9 (56.0 – 67.8)	<0.001				
	Spondyloarthritis^	78.4 (75.1 – 81.7)					
**Spondyloarthritis, Diagnosis**	Psoriatic arthritis					80.2 (75.31 – 85.1)	0.471
	Axial spondyloarthritis					82.4 (78.51 – 86.3)	
	Non-radiographic					64.6 (49.71 – 79.5)	0.001
	Radiographic					84.5 (80.6 – 88.4)	
**Age,** years	<45	79.0 (74.3 – 83.7)	<0.001	55.6 (40.1 – 71.1)	0.037	82.0 (77.1 – 86.9)	0.369
	45 to<65	73.5 (69.6 – 77.4)		41.8 (33.8 – 49.8)		82.4 (78.3 – 86.5)	
	≥65	59.7 (50.9 – 68.5)		35.2 (23.0 – 47.4)		74.5 (62.9 – 86.1)	
**BMI,** kg/m^2^	<30	73.7 (70.2 – 77.2)	0.794	42.9 (35.5 – 50.3)	0.715	81.2 (77.5 – 84.9)	0.728
	≥30	72.8 (67.3 – 78.3)		40.9 (29.5 – 52.3)		82.4 (76.9 – 87.9)	
**Disease duration,** years*	<5	75.0 (70.3 – 79.7)	0.474	42.8 (32.2 – 53.4)	0.079	80.2 (75.1 – 85.3)	0.662
	5 to<10	74.1 (68.0 – 80.2)		51.4 (38.3 – 64.5)		80.7 (74.2 – 87.2)	
	≥10	71.4 (66.7 – 76.1)		37.4 (28.2 – 46.6)		83.5 (78.8 – 88.2)	
**Line of golimumab therapy**	First	83.0 (78.9 – 87.1)	<0.001	54.7 (44.9 – 64.5)	<0.001	87.7 (83.2 – 92.2)	0.001
	Second	73.8 (68.7 – 78.9)		35.1 (23.9 – 46.3)		82.7 (77.6 – 87.8)	
	Third/subsequent	62.4 (56.7 – 68.1)		34.5 (23.9 – 45.1)		73.7 (67.6 – 79.8)	
Methotrexate**	No	72.9 (69.4 – 76.4)	0.623	35.6 (26.6 – 44.6)	0.034	80.5 (77.0 – 84.0)	0.261
	Yes	74.4 (69.3 – 79.5)		48.2 (39.8 – 56.6)		84.6 (78.9 – 90.3)	
Glucocorticoids**	No	77.0 (73.7 – 80.3)	<0.001	47.7 (38.3 – 57.1)	0.193	82.7 (79.4 – 86.0)	0.087
	Yes	64.6 (58.7 – 70.5)		38.4 (30.4 – 46.4)		75.5 (67.1 – 83.9)	
Disease activity***	< median	79.9 (76.0 – 83.8)	0.003	49.3 (39.5 – 59.1)	0.034	87.2 (83.3 – 91.1)	0.018
	≥ median	70.8 (66.3 – 75.3)		35.5 (26.1 – 44.9)		79.6 (74.7 – 84.5)	

Values are retention rates expressed as % (95% confidence interval).

^SpA includes psoriatic arthritis and axial SpA *Disease duration: time since diagnosis to golimumab initiation ** Methotrexate and glucocorticoids as concomitant medication at golimumab initiation. ***Cut-off values used to define disease activity at golimumab initiation above the median were: DAS28 >4.3 (RA), DAS28 >4.2 (peripheral PsA) or BASDAI >5.8 (axial SpA, axial PsA).

BMI, body mass index; CI, confidence interval; RA, rheumatoid arthritis; SpA, spondyloarthritis.

**Table 3:** Cox regression analysis. Factors associated with discontinuation of golimumab when the curve changed from fast to slow decay for a) overall population (month 10), b) rheumatoid arthritis (month 24) and c) spondyloarthropathies (month 8).

	Hazard ratio	95% confidence interval	p
a) Overall population (month 10)			
Diagnosis (RA versus spondyloarthritis^)	1.98	1.43 – 2.74	<0.001
Second versus first biological drug	1.75	1.19 – 2.58	0.005
Third or further versus first biological drug	2.44	1.69 – 3.53	<0.001
Disease activity > median at golimumab initiation*	1.44	1.07 – 1.92	0.015
Corticosteroids at golimumab initiation	1.40	1.00 – 1.93	0.048
b) Rheumatoid arthritis (month 24)			
Age 45 to<65 versus age<45 years	1.77	0.97 – 3.20	0.061
Age ≥65 versus age<45 years	1.81	0.95 – 3.43	0.072
Second versus first biological drug	1.87	1.18 – 2.96	0.008
Third or further versus first biological drug	2.35	1.49 – 3.71	<0.001
Glucocorticoids at golimumab initiation	1.51	1.03 – 2.23	0.036
c) Spondyloarthritis (month 8)^			
Gender (women versus men)	1.64	1.08 – 2.48	0.020
Second versus first biological drug	1.64	0.94 – 2.85	0.083
Third versus first biological drug	2.14	1.25 – 3.69	0.006
Disease activity > median at golimumab initiation*	1.49	0.97 – 2.27	0.066

^ Spondyloarthritis includes axial spondyloarthritis and psoriatic arthritis. *Cut-off values used to define disease activity above the median were: DAS28 >4.3 (RA), DAS28 >4.2 (peripheral PsA) or BASDAI >5.8 (axial SpA, axial PsA).

RA, rheumatoid arthritis.

Biomarkers of inflammation at golimumab initiation (Erythrocyte Sedimentation Rate and C-Reactive Protein) were not associated with golimumab discontinuation in the multivariate analysis. None of the 2-factors interactions analysed showed an HR that was statistically significant with respect to golimumab discontinuation (data not shown).

#### 3.2.2 Rheumatoid arthritis

In RA patients, univariate analysis (**Table 2**) showed that golimumab retention during the initial rapid decline period (at month 24) was significantly higher in younger patients (<45 years), when golimumab was used as first biological therapy, in patients using methotrexate at golimumab initiation, and in patients with disease activity below the median. Cox regression analysis showed that factors associated with golimumab discontinuation at month 24 were the use of golimumab in second (HR 1.87, 95% CI 1.18 - 2.96, p=0.008) or third line (HR 2.35, 95% CI 1.49 - 3.71, p<0.001) compared to first line and treatment with glucocorticoids at golimumab initiation (HR 1.51, 95% CI 1.03 - 2.23, p=0.036). A trend toward discontinuation was observed in patients starting golimumab at age 45 years or older but did not reach statistical significance (**Table 3**). **Table 4** shows the retention rates of golimumab in RA patients at the end of the rapid decay period according to factors categorised by statistical significance in the Cox analysis. The worst retention rates were observed in patients treated with golimumab as third or subsequent line of biological therapy, in combination with glucocorticoids, and aged >65 years (12.2%, 95% CI 0.0 - 27.3); retention rates were higher in biologic naive patients, without glucocorticoids at golimumab initiation, and aged<45 years (85.7%, 95% CI 59.8 - 100).

**Table 4:** Retention rates at the timepoint the curve of golimumab persistence changed from fast to slow decay, stratified by combinations of the variables identified in the Cox analyses.

a) Rheumatoid arthritis (month 24)						
Line of therapy		Glucocorticoids at golimumab initiation		Age (years)		
First (n= 102)	54.7 (44.9–64.5)	No (n= 36)	66.3 (64.8–67.8)	<45 (n= 7)	85.7 (59.8–100)
				45–65 (n= 25)	63.3 (44.1–82.5)
				≥65 (n= 4)	50.0 (1.0–99.0)
		Yes (n= 66)	48.3 (36.0–60.6)	<45 (n= 15)	32.5 (6.8–58.2)
				45–65 (n= 40)	46.5 (30.8–62.2)
				≥65 (n= 11)	72.7 (46.4–99.0)
Second (n= 79)	35.1 (23.9 – 46.3)	No (n= 39)	32.7 (17.2–48.2)	<45 (n= 7)	42.9 (6.2–79.6)
				45–65 (n= 23)	34.8 (14.2–55.4)
				≥65 (n= 9)	22.2 (0–49.4)
		Yes (n= 40)	38.6 (22.5–54.7)	<45 (n= 2)	(*)
				45–65 (n= 29)	38.8 (20.4–57.2)
				≥65 (n= 9)	(**)
Third or subsequent (n= 86)	34.5 (23.9 – 45.1)	No (n= 39)	45.3 (28.6–62.0)	<45 (n= 6)	62.5 (20.8–100)
				45–65 (n= 22)	32.8 (12.0–53.6)
				≥65 (n= 11)	63.6 (35.2–92.0)
		Yes (n= 47)	25.7 (12.6–36.8)	<45 (n= 6)	66.7 (29.1–100)
				45–65 (n= 18)	27.8 (7.0–48.6)
				≥65 (n= 23)	12.2 (0–27.3)
b) Spondyloarthritis (psoriatic arthritis and axial spondyloarthritis) (month 8)						
Line of therapy		Sex		Disease activity		
First (n= 211)	87.7 (83.2–92.2)	Men (n= 130)	91.5 (86.8–96.2)	<median (n= 74)	93.2 (87.5–98.9)
				>median (n= 45)	79.7 (79.7–98.1)
		Women (n= 81)	81.5 (73.1–89.9)	<median (n= 34)	94.1 (86.3–100)
				>median (n= 38)	78.9 (66.0–91.8)
Second (n= 209)	82.7 (77.6 – 87.8)	Men (n= 112)	86.6 (80.3–92.9)	<median (n= 63)	88.9 (81.1–96.7)
				>median (n= 43)	83.7 (72.7–94.7)
		Women (n= 97)	78.3 (70.1–86.5)	<median (n= 34)	76.5 (62.2–90.8)
				>median (n= 50)	78.0 (66.4–89.6)
Third or subsequent (n= 198)	73.7 (67.6 – 79.8)	Men (n= 108)	78.7 (71.1–86.3)	<median (n= 47)	83.0 (72.2–93.8)
				>median (n= 54)	81.5 (71.1–91.9)
		Women (n= 90)	67.7 (58.1–77.3)	<median (n= 30)	80.0 (65.7–94.3)
				>median (n= 40)	64.9 (50.0–79.8)

Values are retention rates expressed as % (95% confidence interval). (*) Only two patients in this group, who did not reach a follow-up of 24 months; (**) This group comprised nine patients, five discontinued golimumab before month 24 and the remaining four were on golimumab therapy but had not reached a follow-up of 24 months at the time of study analysis.

#### 3.2.3 Spondyloarthritis

In patients with SpA (including PsA and ax-SpA), univariate analysis (**Table 2**) showed that golimumab retention during the rapid decline period (at month 8) was significantly higher in men, when golimumab was used as first biological therapy, and in patients with disease activity at golimumab initiation below the median. A trend toward discontinuation was observed in patients treated with glucocorticoids when starting golimumab but did not reach statistical significance (p=0.087). By indication, the retention rate was similar in patients with PsA and axial SpA, although it was poorer in the small group of patients with non-radiographic axial SpA (**Table 2**). Cox regression analysis in this fast decay period (**Table 3**), showed that female sex (HR 1.64, 95% CI 1.08 - 2.48, p=0.020) and the use of golimumab as third/subsequent biologic (HR 2.14, 95% CI 1.25 - 3.69, p=0.006) compared with first line, were factors associated with golimumab discontinuation at month 8. Patients with disease activity higher than the median at golimumab initiation showed a trend to lower retention (p=0.066). The retention rates of golimumab during the rapid decay period according to factors categorised by statistical significance in the Cox regression analysis are shown in **Table 4**. The patient profile with poorer retention rate comprised women starting golimumab as third or subsequent line of biological therapy and with disease activity higher than the median (64.9%, 95% CI 50.0 - 79.8%); retention was higher in biologic naïve men with a disease activity below the median (93.2%, 95% CI 87.5 - 98.9).

## 4 Discussion

The ability to identify the period of rapid loss (i.e., high discontinuation rate) of golimumab under real-world conditions and the factors associated with discontinuation during this early treatment period is the major contribution of the innovative approach used in the current study. Analyses of disease and patient characteristics associated with drug discontinuation in this first rapid loss period can contribute to a better selection of patients most likely to remain on golimumab in the long term. Additionally, we found that the initial high discontinuation rate period observed was, with golimumab, longer for patients with RA (24 months) than with SpA (8 months).

A significant proportion of patients treated with biological DMARDs discontinue therapy early after starting treatment. Retention curves for patients taking biologics show that most discontinuations occur in the first months of treatment, after which the discontinuation rate slows (10–13). The ability to characterise the patients most likely to discontinue a biologic during this early period can help to better select the most appropriate compound for specific patient profiles, increasing the likelihood of achieving a sustained long-term response, improving patients’ clinical outcomes and likely reducing costs.

In this study, an exponential two-phase decay model was used to determine the time point at which golimumab retention curves shifted from high to low discontinuation rates; an analysis that, to our knowledge, has not previously been used in studies with TNFi. For the entire population, the curve shifted from a fast to a slow decay at month 10 (retention rate at that point, 73.4%). An interesting finding is that the rapid decay period was longer in RA, 24 months (retention rate 45.0%) than in SpA patients, 8 months (retention rate 81.6%), indicating that early discontinuation of golimumab in RA accounts for the lower 8-year retention rate observed (24.6% in RA versus 41.9% in SpA). The complexity of RA, more stringent clinical goals such as achieving remission or low disease activity, comorbidities, and the greater number of targeted therapies available (biological drugs and targeted synthetic DMARDs) may contribute to the lower short- and long-term retention rates observed (20). Several reports have highlighted the differences in the mid- and long-term retention rates of anti-TNF therapies in RA and SpA patients (21, 22). In SpA, a recent study with secukinumab (an interleukin 17A inhibitor) in patients with ax-SpA and PsA patients using the same methodology (n = 138 patients and maximum follow-up period of 5 years) identified month 12 as the timepoint the curve changed from fast to slow decay, and diagnosis, obesity, and gender as relevant variables: the best retention rates were seen in the groups of women with ax-SpA and men with PsA (13).

In our study, factors associated with golimumab discontinuation in the overall population during the high discontinuation rate period (first 10 months) were RA diagnosis, previous exposure to biologics, disease activity above the median, and treatment with glucocorticoids at golimumab initiation. By treatment indication, in RA patients, factors significantly associated with golimumab discontinuation during the fast decay period of 24 months included use of golimumab after prior biological therapy and in combination with glucocorticoids, with a trend toward poorer retention with increasing age. In the SpA cohort, factors associated with discontinuation at month 8 (when the rapid decay period ended) were use of golimumab as second or third/subsequent line of biological therapy, disease activity higher than median at golimumab initiation, and female sex. Previous reports, although not focussed on analysis of the initial period of rapid loss, have found factors associated with poor retention of biologics, including golimumab, consistent with those of our study. A Spanish study evaluating several biologics found that a greater proportion of ax-SpA and PsA patients remained on their first drug after 4 years of follow-up compared to RA patients (23). Our finding of a lower retention rate of golimumab in patients with non-radiographic ax-SpA compared to the radiographic form must be interpreted cautiously, as it comes from a very small group of patients (n= 40). There seems to be agreement that truly non-radiographic ax-SpA represents an early stage or abortive type of the radiographic form (24), but we could not provide long-term retention in the non-radiographic group because the approval of golimumab for use in this early stage was recent and patients did not have a long enough follow-up period. That the later lines of biologics were associated with a higher risk of discontinuation has been previously reported not only for golimumab (25, 26), but also for other biologics, including TNFi (27–30).

Use of glucocorticoids at golimumab initiation was associated with early discontinuation in RA but not in SpA in our cohort. The difference in the frequency of use of these therapies (57.3% in RA and only 16.5% in SpA in our cohort) may account for this difference. Treatment with glucocorticoids may reflect more severe, treatment-resistant disease and has previously been identified as a predictor of discontinuation of biologic therapies in RA due to lack of efficacy (31), but glucocorticoids can also reduce DAS 28. This might account for the lack of significant association between disease activity in RA and discontinuation of golimumab. In univariate analysis, we observed no differences in retention between men and women in RA (with 77% being women). However, in SpA patients, a higher discontinuation rate was observed in women, a finding that is consistent with previous reports. Women can experience more severe disease activity, higher levels of pain, or greater functional impairment compared to men, leading to more frequent changes in treatment, although limited research has addressed the reasons for this finding (32, 33).

The main study objective was to evaluate whether identifying factors associated with initial rapid loss of golimumab could help select patients with a higher likelihood of long-term retention of this biological agent. A previous study showed that in patients with RA and PsA treated with biologics, mainly TNFi in the first indication, persistence at 1 year and low activity at that time predicted persistence at 12 years (5). In that study, the 1-year time point was chosen for convenience and not, as in our case, after an analysis of trend changes in drug retention. Interestingly, in a recently published golimumab study using the same data set from the BIOBADASER registry but focusing on 8-year probability of retention (10), it was observed that factors positively associated with long-term retention of golimumab in the overall cohort included its use as first-line biologic therapy, diagnosis of SpA (vs. RA), and concomitant treatment with methotrexate, while factors associated with higher discontinuation rates were glucocorticoid use and higher than median disease activity. In the same study (10), in RA patients, older age, glucocorticoid therapy, and higher than median disease activity at golimumab initiation were associated with a higher likelihood of discontinuation of golimumab during the 8-year follow up. In ax-SpA, female gender and higher than median disease activity at golimumab initiation, and in PsA, use of biologics in non-naive patients, were associated with higher odds of golimumab discontinuation. Notably, these results, obtained from an 8-year long-term analysis, identified factors associated with golimumab discontinuation that are highly consistent with factors identified in the present study using early discontinuation data analysis. The practical implication of these findings is that an early analysis of retention rates and associated factors might help in selecting patient profiles most likely to respond in the long term to a certain therapy which, ultimately, might have positive clinical and economic implications.

Retention rates based on the factors found in the Cox regression analysis (**Table 4**) may be useful in the decision-making process when initiating targeted therapy. The worst retention rates were observed in RA patients treated with golimumab as a third and subsequent line of biologic therapy in combination with glucocorticoids and who were older than 65 years, in whom another therapeutic option could be considered. While retention rates in SpA patients were generally high, patients with the best retention rates were men with SpA treated with golimumab as first biological drug and with disease activity below the median at the initiation of golimumab. Similar studies with other biological DMARDs would help to clarify the benefit of specific therapies for specific patient profiles.

Our study has the inherent limitations of observational and registry studies, such as the lack of information on dosing strategies, other concomitant medications, and other potential confounders such as skin involvement (in PsA patients) or degree of joint damage that could affect treatment persistence. Stratification by the different variables resulted in a low sample size in several subgroups; thus, the retention figures displayed must be interpreted cautiously. This was an analysis restricted to patients treated with golimumab, but future studies, either with other individual biological or targeted synthetic DMARDs or comparisons between different therapeutic agents, can contribute to identifying patient profiles that can benefit from different agents, which will be very helpful to make informed decisions. Strengths include the use of routine clinical practice data, reflecting a real-world patient population, and the long follow-up period.

In summary, with this innovative analysis, we have identified patient profiles that are most likely to discontinue treatment early and, consequently, those most likely to remain on long-term golimumab. This information may facilitate earlier selection of patients expected to have better long-term retention on golimumab. Studies like the present one, conducted with other TNFi and/or drugs with different mechanisms of action, should be able to provide additional information for the optimal selection of advanced therapies.

## Data availability statement

The original contributions presented in the study are included in the article/supplementary materials, further inquiries can be directed to the corresponding author.

## Ethics statement

The study was performed in accordance with Good Pharmacoepidemiology Practice standards, the principles of the Declaration of Helsinki, and with Spanish regulations on data protection and research. Patients provide informed consent prior to enrolling in BIOBADASER, which includes consent for subsequent analysis of anonymised aggregated data, as approved by the Clinical Research Committee of the Hospital Universitario Clinic Barcelona (code FER-ADA-2015-01). Consequently, specific informed consent for the current analysis was not needed. The studies were conducted in accordance with the local legislation and institutional requirements. The participants provided their written informed consent to participate in this study. Written informed consent was obtained from the individual(s) for the publication of any potentially identifiable images or data included in this article.

## Author contributions

AG-D: Writing – original draft, Writing – review & editing. EG-D: Writing – original draft, Writing – review & editing. MS-J: Writing – original draft, Writing – review & editing. LC-C: Writing – original draft, Writing – review & editing. MP-S: Writing – original draft, Writing – review & editing. FS-A: Writing – original draft, Writing – review & editing. IC: Writing – original draft, Writing – review & editing. FD-G: Writing – original draft, Writing – review & editing.
